# Incorporating Medical Students Into Primary Care Telehealth Visits: Tutorial

**DOI:** 10.2196/24300

**Published:** 2021-05-28

**Authors:** Aanika Balaji, Sarah Lou Clever

**Affiliations:** 1 Johns Hopkins University School of Medicine Baltimore, MD United States; 2 Department of Medicine Johns Hopkins University Baltimore, MD United States

**Keywords:** medical student, education, primary care, telehealth, video visits, internal medicine, medical education, teleconsultation, digital health, COVID-19

## Abstract

**Background:**

The COVID-19 pandemic has brought about sweeping change in health care delivery, which has shifted from in-person consultations to a web-based format. Few medical schools provide web-based medicine or telemedicine training to their learners, though this is likely to be important for future medical practice.

**Objective:**

This tutorial communicates a framework for incorporating medical students into primary care telemedicine clinics.

**Methods:**

A third-year medical student and internal medicine attending physician from the Johns Hopkins University completed telemedicine clinic visits in April 2020 by using a variety of video platforms and via telephone calls.

**Results:**

Nine telemedicine visits were completed over 4 clinic days. Our patients were, on average, aged 68 years. The majority of patients were female (6/9, 67%), and most appointments were completed via a video platform (6/9, 67%). Additionally, our experience is summarized and describe (1) practical tips for how to prepare for a telehealth visit; (2) technology considerations; (3) recommendations for participation during a telehealth visit; (4) debriefing and feedback; (5) challenges to care; and (6) student, care provider, and patient reactions to telemedicine visits.

**Conclusions:**

Telemedicine clinics have been successfully used for managing patients with chronic conditions, those who have attended low-risk urgent care visits, and those with mental health concerns. Patients have reported high patient satisfaction scores for telemedicine visits, and the majority of patients are comfortable with having medical students as part of their care team. Moving forward, telemedicine will remain a popular method for receiving health care. This study has highlighted that medical students can successfully be integrated into telemedicine clinics and that they should be exposed to telehealth whenever possible prior to residency.

## Introduction

The COVID-19 pandemic era is a historic moment that is ushering in waves of challenges and the need for innovation. As a society, we have had to adapt to wearing face masks, working from home, and practicing social distancing measures to prevent the further spread of SARS-CoV-2 [1]. In alignment with these recommendations, the Association of American Medical Colleges requested a temporary suspension of medical student involvement in on-site clerkships that involve direct patient contact between March and April 2020 [2]. Almost overnight, medical schools and health care systems had to shift from in-person learning and appointments to a web-based format.

Defined as the use of telecommunication and electronic information to promote long-distance health care among patients and care providers, telemedicine is well suited to fill this gap. The practice of telemedicine is relatively new; its expanded use began in the 2010s [3,4]. In spite of this increased amount of use, a review by Pourmand et al [5] highlighted that nearly 40% of medical schools did not offer any formal instruction in telemedicine as part of their curriculum in the 2017-2018 academic year. Without a standardized curriculum or learning objectives, medical schools and residency programs have independently adapted and created new web-based clerkships and telemedicine electives for medical students and trainees during the pandemic [6-8]. These web-based experiences provide opportunities for advancing students’ clinical education but often have either limited or no patient interaction components [9]. However, there is no literature that informs clinicians and medical students about how to participate in telehealth visits in the primary care setting.

To address this gap in knowledge, this tutorial aims to provide a summary of experiences, methods, the lessons learned about telehealth from both the student and attending physician perspective, and a framework for incorporating future learners into telemedicine clinics.

### Background

As in-person clinical clerkships were suspended for 2 months at the Johns Hopkins University School of Medicine (March to May 2020), students were able to enroll in web-based learning offerings. However, these courses did not involve patient interaction. Prior to the COVID-19 pandemic–related suspension of clinical clerkships, the coauthors (an attending internal medicine physician [SC] and third-year medical student [AB]) had begun working together as part of an elective primary care clerkship while AB completed her Master of Public Health program. AB had attended 4 in-person clinic sessions and, by the last clinic session, had been interviewing patients independently and reporting to SC. In March 2020, SC’s clinic was converted to a fully remote, video visit–only clinic [10]. In April 2020, SC decided to try incorporating AB into the web-based clinic. The experience is summarized below.

### Telehealth Visits: A Practical Guide

#### Prior to the Start of Web-Based Visits

**Figure 1:**
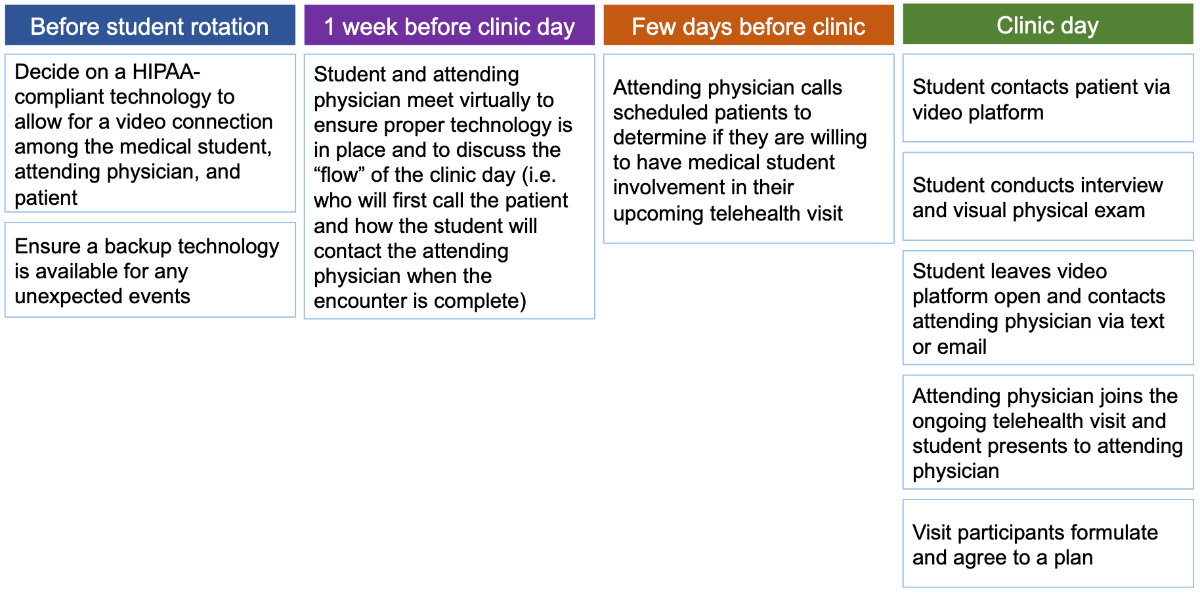
Flowchart of steps for including medical students in telehealth. The flowchart is organized by the time periods before a clinic day. HIPAA: Health Insurance Portability and Accountability Act.

SC and AB met via Zoom (Zoom Video Communications Inc) prior to the first clinic day to discuss the new clinic format, review expectations and objectives (Textbox 1), and practice navigating options for patient communication. It was agreed that SC would contact her patients prior to the clinic day to inquire whether they were comfortable with having a medical student start the visit, and AB would prepare for visits by reviewing each patient’s medical record. As they had previously worked together, SC was comfortable with having AB connect to patients and start visits on her own for 10 to 15 minutes before SC joined the web-based room to hear AB present the interval history and jointly create an assessment and plan. They had decided that all communication about the patient would occur while the patient was present “in the room,” as had been done in prior in-person visits. This information, and that which follows, is presented as a flowchart in Figure 1.

**Outlined objectives and expectations for medical students attending telehealth clinics. These objectives were adapted from the Genes to Society Longitudinal Clerkship Curriculum from the Johns Hopkins School of Medicine.**

**Medical student expectations and objectives from telehealth clinic**

- Experience the clinical practice of telehealth in a primary care setting through interviewing patients
- Assist in managing chronic disease in a telehealth setting
- Learn about how illness may be impacted by the COVID-19 pandemic
- Foster clinical skills by delivering ambulatory telehealth care
  - Review a patient’s medical record prior to the visit
  - Conduct a focused interview
  - Gather objective clinical data in lieu of an in-person physical exam
  - Formulate an appropriate assessment and plan
  - Communicate information to both care providers and patients

#### Technology

The Johns Hopkins health system uses Epic (Epic Systems Corporation), which has Polycom Telecommunications (Polycom Inc) built in as the default web-based visit communication app. This service accommodates up to 4 users, thereby allowing a medical student, patient, and attending physician to be present on the same screen during the visit. All users must download the Polycom app onto their computers, tablets, or phones and have access to both video cameras and microphones for communication. Polycom does not support direct messaging among parties. Recently, our hospital’s Epic system has transitioned to using WebEx, a video platform hosted by Cisco that has an interface and functionalities that are similar to those of Polycom in terms of video visits. Messages between patients and care providers had to be sent through MyChart, a secure messaging platform hosted on Epic.

Another option for video visits was the Doximity app (Doximity Inc). Doximity can be used to conduct video visits with 3 users via cell phones or a desktop browser. When the care provider starts a video call, the patient receives a text message to join a secure video room via their cell phone’s internet browser. A third user can be added by the provider who started the call. Doximity calls do not support synchronous messaging.

Zoom technologies can be used as well but were not explored for this clinical elective, as they were not approved for use in clinical encounters by our institution. Zoom meeting links must be password-protected to be used. All audio, video, and screen sharing data are encrypted, and the platform does not have access to identifiable patient health information. This service can host multiple parties and has synchronous messaging capabilities.

If patients are not able to join the visit via video link, the Doximity dialer was used by AB or SC to call patients from their private phones. This app displays the clinic’s telephone number on the patient’s phone, not the provider’s personal phone number. A third party can be added to the call line.

All of the apps discussed are Health Insurance Portability and Accountability Act compliant and use encryption methods so that both videos and messages between patients and care providers are secure [11]. Zoom technologies, Cisco technologies (WebEx), and Doximity all use the Advanced Encryption Standard (AES) with 256-bit keys to encrypt meetings [12-14]. Polycom uses the AES with 128-bit encryption [15].

#### During the Visit

Before beginning the clinic day, both SC and AB were in private rooms in their homes to ensure patient confidentiality. These rooms provided neutral backgrounds and adequate lighting. Both SC and AB used headphones to prevent patient conversations from being heard by others if other people were present in their homes.

For each visit, AB and SC called patients 10 to 15 minutes before the visit start time. SC briefly introduced herself, explained her role during the visit, and provided context for why visits had shifted to a web-based format before exiting the meeting. Each visit was scheduled for 30 minutes. She gathered information about patients’ reasons for the visit, their interval history, and performed a brief physical exam (appearance, mental state, and skin exams, if appropriate). Some patients recorded their blood glucose or blood pressure readings and, if they were relevant to their medical histories, AB collected these data. A handful of patients had physical exam findings captured on their cell phones as photos (eg, skin rashes), which they were able to share by presenting their phones to the webcam.

AB wrote progress notes in each patient’s electronic medical record and pended relevant orders during visits. After 15 minutes, SC rejoined the room and AB provided a brief patient presentation as well as her initial thoughts. Then, SC gathered more relevant data, and with the patient’s input, all three participants discussed the next steps for the patient’s care.

#### Debriefing and Feedback

After the last patient visit, AB and SC debriefed quickly to go over notes and what orders needed to be placed. AB completed progress notes within 1 hour after the visit. Afterward, she and SC conducted a longer (about 30 minutes) call to go over further feedback.

During the feedback call, which mirrored the feedback provided during an in-person clinical session, SC and AB discussed each patient visit to highlight teaching points and offer feedback to AB. A summary of the guidelines from this section is provided and outlined in Textbox 2.

**Tips for incorporating medical students into primary care telehealth visits collected over 4 telehealth sessions. The tips are subdivided into the following categories: (1) prior to the telehealth visit, (2) technology considerations, (3) during the telehealth visit, and (4) debriefing and feedback.**

**Prior to the telehealth visit**

- Decide what role the student will play during the visit (shadowing vs completing part or all of the web-based visit)
- Have the attending physician or a medical assistant reach out to patients to obtain permission for students to be a part of their care
- Frequent communication between the medical student and attending physician before, during, and after telemedicine visits is recommended.

**Technology considerations**

- Have a back-up plan if the first video communication platform does not work
- Use Doximity to mask outgoing phone numbers if communicating via phone

**During the telehealth visit**

- Conduct each visit in a quiet, private room to protect patient confidentiality
- Ensure that the patient is in a quiet, secure location at the beginning of the interview
- Describe the student’s role in the patient’s visit
- Student may exit the web-based clinic room if all materials have been covered prior to the attending physician’s return
- Student may complete the patient presentation while in the web-based room

**Debriefing and feedback**

- Set time aside after each clinic day to provide timely and constructive feedback.
- The medical student can collect questions about patients and discuss them with the attending physician during this time.

### Visit Characteristics

Over the course of 4 telehealth clinic sessions, SC and AB interviewed 9 patients. Patients were included if (1) their visits coincided with the clinic days when AB was able to join SC and (2) they were amenable to having a medical student involved in their care. Most appointments were completed via a video platform (6/9) instead of via telephone (3/9). Of the 9 appointments, 6 were annual or routine checkups and 3 were problem-focused visits (blood pressure, diabetes medication change, and posthospital discharge visits). At the time of writing, Maryland had mandated a stay-at-home order, and only urgent visits requiring in-person services were conducted in the office. None of the 9 patients we saw were invited for further in-person follow-ups after their initial appointment.

### Challenges to Care

During this outpatient elective, a few challenges arose that were unique to telemedicine. Physical exams in telemedicine consults are limited to visual inspections and verbal interactions. One patient had a rash on his leg, and while it was initially difficult to share the image he had captured on his phone, he was able to align both screens to provide the team with a clear view. Based on the image and his history of present illness, we offered conservative topical therapy and advised that if symptoms worsen, he would need to follow up with the dermatology department. Relying on visual inspection for more complex diagnoses can be challenging and may not be feasible through telemedicine alone. Perkins et al [16] described in their letter to the editor how their practice has conducted teledermatology visits—they relied on patients taking multiple high-resolution images and uploading them to MyChart (a patient portal that provides access to patients’ medical records and facilitates communication with care providers) before their appointments. These hybrid approaches (ie, combining stored data with synchronous visits) can result in better, informed, visual physical exams and evaluations of patient concerns. Other studies have reported using guides for having patients conduct a self-physical exam either alone or with a partner [17].

A third (3/9, 33%) of the patients seen had conducted their visits via telephone, as patients were unable to troubleshoot their video connections. This means of communication further limits the physical examination of patients and increases the difficulty of building rapport with patients. However, for noncomplicated visits, the medical team was able to triage patient health concerns, reorder medications, and provide health counseling without difficulty.

SC and AB relied on patients to provide their own health data. This became important for focused follow-up visits in which blood pressure or blood glucose were monitored. These data are limited by patients’ ability to use home health care devices and the accuracy of the devices themselves. The medical team did not have the capability at the time to validate self-reported data through home nursing or to invite patients to the clinic for blood pressure or point-of-care blood glucose tests. At the time of writing, the outpatient elective coincided with a state-mandated stay-at-home order issued by Maryland. Therefore, patients were only offered in-person consultations if they had urgent symptoms and were not routinely seen in person for follow-ups.

### Reactions to Telemedicine Visits

#### Student Perspective

At the time of this clerkship, it was unclear when medical students would be able to return to the wards. AB found that this telemedicine elective added value to her medical education, thereby allowing her to further hone her skills in understanding the patient history, formulating a differential diagnosis, and creating an appropriate assessment and plan. She learned to quickly build rapport with patients over web-based platforms, set an agenda, and adequately discuss health concerns. Similar to an in-person rotation, AB was able to present each patient case to the attending physician of her patients, pend orders, write clinical notes, and receive real-time feedback from the supervising physician. Importantly, while telemedicine is a step removed from physically seeing and touching patients, it provided the safest alternative during the COVID-19 pandemic that still emphasizes learning with and from patients. AB did not have formal training in telemedicine prior to this elective. She realized that training in telemedicine is a skill set that will be useful and necessary in the postpandemic world, especially for follow-ups that involve discussing results or conducting psychiatric-focused visits.

#### Attending Physician Perspective

SC was eager to include a student in telehealth visits, as it seemed clear that determining how to do so would be necessary to continue the meaningful education of medical students in outpatient care delivery during and after the pandemic. AB and SC were able to navigate the technology prior to the visits well enough that SC was confident that they could be successful in providing care and medical education at the same time. SC was concerned that contacting patients before the visits might be overly burdensome but found that it was not. Since AB was able to start the visits early, there was enough time for her to present patients to SC and keep within the visit time. SC’s patients seemed to genuinely enjoy having a student involved in their care, and SC appreciated the opportunity to return to teaching during such a stressful time.

#### Patient Perspective

This study did not collect postvisit feedback from patients; therefore, the patient perspective was gleaned from interactions during visits and a review of the literature. All patients agreed to have a medical student as part of their care. Patients had extended appointment times, as the medical student started the visit early and the attending physician joined after 15 minutes. Additionally, patients were able to hear their visit presentation and add or clarify information. A survey of outpatients by Simons et al [18] showed the majority of patients are comfortable with medical students being involved in their care if permission was sought beforehand, they knew the role of the medical student, time limits were set, and patients were able to speak with the attending physician. Other studies have confirmed these findings—patients reported having more time with the care team and found that it was beneficial to have medical students involved in their care [19,20].

## Discussion

The COVID-19 pandemic has highlighted that for some medical needs, such as managing patients with chronic conditions or mental health concerns and those who have attended low-risk urgent care visits, telehealth has successfully provided patients with a socially distanced means to receive care [21,22]. Although there is a loss of in-person connection, this method of care delivery provides both patients and care providers with the opportunity to connect without the need for personal protective equipment while decreasing the burden of travel for all participants, and the ability to receive and deliver medical care in a safe, comfortable environment. Early studies have reported that patient satisfaction scores for primary care and family practice telemedicine appointments were comparable to those for in-person visits [23]. Importantly, these data indicate that telemedicine is a successful alternative to in-person visits, especially during the COVID-19 pandemic [24].

From the learner perspective, telehealth visits do not fully replace the experience and education of seeing patients in clinics, such as the experience of completing physical exams and appreciating both normal and abnormal findings. Frequent and ample communication between an attending physician and student facilitated real-time discussions about patient health concerns, troubleshooting technology, and methods for improving visits with patients. Telemedicine has a valuable role in medical education and is an essential skill for the modern medical student [6,25].

This tutorial aims to provide practical advice from both the student and attending physician perspective to successfully integrate medical students into telehealth clinics. Medical students must be exposed to this method of care delivery prior to residency, and their practice can start now [26].

## Abbreviations

AES: Advanced Encryption Standard
